# Developing as an independent practitioner in psychiatry

**DOI:** 10.1192/j.eurpsy.2023.196

**Published:** 2023-07-19

**Authors:** J. Wise

**Affiliations:** Brent, CNWL, London, United Kingdom

## Abstract

**Abstract:**

Dr Wise will help the audience familiarise themselves with the attitudes, atributes and skills neccessary to establish and maintain a practice outside of a state-supported environment, to develop and grow a career, and reduce the risk of burn out.

**Disclosure of Interest:**

None Declared

